# Drug–drug interaction signals between loop diuretics and teicoplanin during acute kidney injury evaluated using Japanese spontaneous adverse drug event reports

**DOI:** 10.1038/s41598-023-41095-4

**Published:** 2023-08-26

**Authors:** Toshinori Hirai, Yuki Kondo, Yuka Sakazaki, Ayaka Seki, Yoichi Ishitsuka, Takuya Iwamoto

**Affiliations:** 1 Department of Pharmacy, Faculty of Medicine, Mie University Hospital, Mie University, 2-174 Edobashi, Tsu, Mie 514-8507 Japan; 2 Department of Clinical Chemistry and Informatics, Graduate School of Pharmaceutical Sciences, Kumamoto University, 5-1 Oehonmachi, Chuo-ku, Kumamoto, Kumamoto 862-0973 Japan

**Keywords:** Medical research, Nephrology, Risk factors

## Abstract

Teicoplanin can cause acute kidney injury, but little is known about the risk of acute kidney injury when teicoplanin is co-administered with loop diuretics (a powerful diuresis), which can alter renal hemodynamics and glomerular filtration rate. We performed a signal detection analysis using a Japanese adverse event database to determine the additive impact of loop diuretics on acute kidney injury associated with teicoplanin. The dataset originated between April 2004 and August 2022. Disproportionality analysis was performed to detect the signals for acute kidney injury (the Standardized MedDRA Query) when co-administered teicoplanin or vancomycin (a positive control) with individual diuretics, including loop diuretics. Multivariate logistic regression analysis was tested to estimate the adjusted reporting odds ratio (aROR) and 95% confidence interval (95% CI). There were 147 and 515 events of acute kidney injury associated with teicoplanin and vancomycin, respectively. A significant positive signal for acute kidney injury when teicoplanin was co-administered with loop diuretics was present (aROR 4.83, 95% CI 3.52–6.61, p < 0.0001). Contrastingly, no significant signals were observed when vancomycin was co-administered with any diuretics. These findings suggest that co-administered loop diuretics may have an unfavorable effect on acute kidney injury while undertaking teicoplanin but not vancomycin.

## Introduction

Glycopeptide antibiotics, such as vancomycin and teicoplanin, are effective at inhibiting the cell wall synthesis of gram-positive bacteria, including methicillin-resistant *Staphylococcus aureus*^1^. Several systematic reviews have shown that teicoplanin is non-inferior to vancomycin in treating serious infections such as bacteremia caused by methicillin-resistant *Staphylococcus aureus*^2,3^. However, glycopeptide antibiotics have a concentration-dependent antibacterial effect with a narrow therapeutic window^4,5^, and their total clearance is linearly correlated with the glomerular filtration rate^6,7^.

Teicoplanin is associated with a lower incidence of acute kidney injury compared with vancomycin^3^. Our recent meta-analysis identified hypoalbuminemia as a potential risk factor for acute kidney injury associated with teicoplanin^8^. However, it remains unclear whether co-administered medications can also increase the risk of acute kidney injury, as most studies have not collected data on nephrotoxic drugs such as diuretics^8^. Diuretics, in particular, consistently reduce the glomerular filtration rate in both healthy volunteers and patients with cardiac failure^9^. The removal of a large amount of fluid by diuretics might increase the risk of acute kidney injury associated with teicoplanin. We hypothesized that the co-administration of teicoplanin and loop diuretics, which have a powerful diuretic effect, could increase the risk of acute kidney injury.

The current study aimed to conduct a signal detection analysis of drug-drug interactions using the Japanese Adverse Drug Event Report (JADER) database to confirm the involvement of diuretics in acute kidney injury associated with teicoplanin referring to vancomycin.

## Results

### Study population

**Figure 1:**
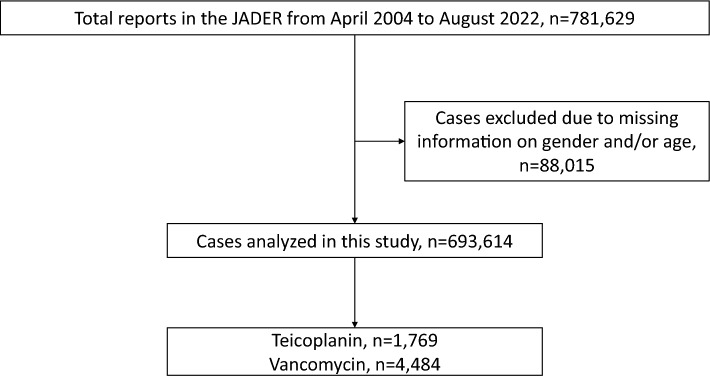
Flowchart for selection of study population. A total of 781,629 cases were identified in the JADER database. After excluding 88,015 cases, we analyzed the remaining cases (n = 693,614). *JADER* Japanese Adverse Drug Event Report.

Figure 1 presents a flowchart summarizing the process for selecting the study population. The dataset contained 781,629 reports between April 2004 and August 2022. After data cleaning, we excluded 88,015 reports due to incomplete data and used 693,614 reports for further analysis.

Table 1 shows the clinical characteristics of patients with acute kidney injury associated with teicoplanin and vancomycin. In the teicoplanin and vancomycin groups, 90 (61.2%) and 331 (64.3%) patients were males, respectively. Approximately 40% of patients in both groups were elderly (above 70 years of age). The teicoplanin group had 66 (44.9%) reports of severe (death or no recovery) acute kidney injury, while the vancomycin group had 161 (31.3%) reports of severe acute kidney injury.

**Table 1:** Clinical characteristics of the study population who developed acute kidney injury associated with teicoplanin or vancomycin.

	Teicoplanin, n (%)	Vancomycin, n (%)
**Sex**		
Male	90 (61.2%)	331 (64.3%)
Female	57 (38.8%)	184 (35.7%)
**Age, years**		
< 10	11 (7.5%)	19 (3.7%)
10–19	10 (6.8%)	8 (1.6%)
20–29	5 (3.4%)	24 (4.7%)
30–39	6 (4.1%)	41 (8.0%)
40–49	9 (6.1%)	52 (10.1%)
50–59	14 (9.5%)	69 (13.4%)
60–69	28 (19.0%)	106 (20.6%)
70–79	38 (25.9%)	110 (21.4%)
80–89	18 (12.2%)	73 (14.2%)
90–99	8 (5.4%)	13 (2.5%)
> 100	0 (0%)	0 (0%)
**Severity**		
Death	26 (17.7%)	65 (12.6%)
No recovery	40 (27.2%)	96 (18.6%)
After–effect	3 (2.0%)	8 (1.6%)
Remission	31 (21.1%)	138 (26.8%)
Recovery	33 (22.4%)	142 (27.6%)
Unknown	14 (9.5%)	66 (12.8%)
**Co-existing diseases**		
Chronic kidney disease	8 (5.4%)	16 (3.1%)
Hypertension	9 (6.1%)	41 (8.0%)
Diabetes	10 (6.8%)	58 (11.3%)
Cardiac failure	10 (6.8%)	33 (6.4%)
Sepsis	13 (8.8%)	54 (10.5%)

Data are presented as numbers (%).

### Signal detection for acute kidney injury

Table 2 presents the findings of the disproportionality analysis for acute kidney injury associated with teicoplanin. After adjusting for relevant variables, we found a significant positive signal only in teicoplanin with loop diuretics (adjusted reporting odds ratio [aROR] 4.83, 95% confidence interval [95% CI] 3.52–6.61). We did not observe significant signals in teicoplanin with thiazide diuretics (aROR 1.18, 95% CI 0.15–9.20), potassium-sparing diuretics (aROR 3.62, 95% CI 2.01–6.51), or vaptans (aROR 6.34, 95% CI 1.67–24.0). We were unable to analyze the signal for thiazide-like diuretics, as there were no reported events. Conversely, we did not observe significant signals of acute kidney injury in vancomycin with individual diuretics (Table 3). The summary of area under the curve shows in Supplementary Table 1.

**Table 2:** Disproportionality analysis for acute kidney injury associated with teicoplanin and diuretics.

	AKI	Without AKI	Crude ROR	95% CI	Adjusted ROR	95% CI	p-value
**Loop diuretics**							
TEIC	100	1338	2.34	1.91–2.87	2.37	1.93–2.91	< 0.0001
Loop diuretics	2386	29,727	2.52	2.41–2.63	1.87	1.78–1.97	< 0.0001
TEIC + loop diuretics	47	284	5.19	3.81–7.07	4.83	3.52–6.61	< 0.0001
Non-target drugs	20,392	639,340	1.00	–	1.00	–	–
**Thiazide diuretics**							
TEIC	146	1606	2.71	2.29–3.22	2.68	2.25–3.19	< 0.0001
Thiazide diuretics	597	7140	2.50	2.29–2.72	1.74	1.60–1.90	< 0.0001
TEIC + thiazide diuretics	1	16	1.87	0.25–14.1	1.18	0.15–9.20	0.8721
Non-target drugs	22,181	661,927	1.00	–	1.00	–	–
**Thiazide-like diuretics**							
TEIC	147	1622	2.67	2.25–3.16	2.64	2.22–3.14	< 0.0001
Thiazide-like diuretics	111	1401	2.33	1.92–2.83	1.59	1.31–1.94	< 0.0001
TEIC + thiazide-like diuretics	0	0	–	–	–	–	–
Non-target drugs	22,667	667,666	1.00	–	1.00	–	–
**Potassium-sparing diuretics**							
TEIC	134	1522	2.68	2.24–3.19	2.67	2.23–3.20	< 0.0001
Potassium-sparing diuretics	1192	13,017	2.78	2.62–2.96	2.13	1.99–2.27	< 0.0001
TEIC + potassium-sparing diuretics	13	100	3.95	2.22–7.04	3.62	2.01–6.51	< 0.0001
Non-target drugs	21,586	656,050	1.00	–	1.00	–	–
**Vaptans**							
TEIC	144	1613	2.66	2.24–3.16	2.63	2.21–3.14	< 0.0001
Vaptans	429	3107	4.11	3.72–4.56	2.47	2.21–2.75	< 0.0001
TEIC + vaptans	3	9	9.93	2.69–36.7	6.34	1.67–24.0	0.0066
Non-target drugs	22,349	665,960	1.00	–	1.00	–	–

The number of patients with and without AKI in each category was presented. The target drugs were TEIC with intravenous administration and diuretics. A multivariate logistic regression model was used to determine the adjusted ROR by introducing age, sex, reporting year, and co-existing diseases (chronic kidney disease, hypertension, diabetes, cardiac failure, and sepsis).

*AKI* acute kidney injury, *ROR* reporting odds ratio, *95% CI* 95% confidence interval, *TEIC* teicoplanin.

**Table 3:** Disproportionality analysis for acute kidney injury associated with vancomycin and diuretics.

	AKI	Without AKI	Crude ROR	95% CI	Adjusted ROR	95% CI	p-value
**Loop diuretics**							
VCM	432	3312	4.14	3.75–4.59	3.97	3.57–4.40	< 0.0001
Loop diuretics	2350	29,354	2.54	2.43–2.66	1.90	1.81–2.00	< 0.0001
VCM + loop diuretics	83	657	4.01	3.19–5.05	3.61	2.86–4.55	< 0.0001
Non-target drugs	20,060	637,366	1.00	–	1.00	–	–
**Thiazide diuretics**							
VCM	514	3924	3.96	3.61–4.35	3.73	3.39–4.11	< 0.0001
Thiazide diuretics	597	7111	2.54	2.33–2.76	1.77	1.62–1.93	< 0.0001
VCM + thiazide diuretics	1	45	0.67	0.09–4.88	0.52	0.07–3.82	0.5241
Non-target drugs	21,813	659,609	1.00	–	1.00	–	–
**Thiazide-like diuretics**							
VCM	514	3961	3.87	3.53–4.25	3.67	3.33–4.04	< 0.0001
Thiazide-like diuretics	110	1393	2.36	1.94–2.86	1.61	1.32–1.96	< 0.0001
VCM + thiazide-like diuretics	1	8	3.73	0.47–29.8	1.66	0.20–13.8	0.6370
Non-target drugs	22,300	665,327	1.00	–	1.00	–	–
**Potassium-sparing diuretics**							
VCM	483	3742	3.97	3.61–4.37	3.77	3.41–4.16	< 0.0001
Potassium-sparing diuretics	1173	12,890	2.80	2.64–2.98	2.14	2.01–2.29	< 0.0001
VCM + potassium-sparing diuretics	32	227	4.34	3.00–6.29	3.96	2.72–5.76	< 0.0001
Non-target drugs	21,237	653,830	1.00	–	1.00	–	–
**Vaptans**							
VCM	510	3946	3.90	3.55–4.28	3.70	3.36–4.07	< 0.0001
Vaptans	427	3093	4.17	3.76–4.62	2.51	2.25–2.80	< 0.0001
VCM + vaptans	5	23	6.56	2.49–17.3	4.18	1.57–11.1	0.0042
Non-target drugs	21,983	663,627	1.00	–	1.00	–	–

The number of patients with and without AKI in each category was presented. The target drugs were VCM with intravenous administration and diuretics. A multivariate logistic regression model was used to determine the adjusted ROR by introducing age, sex, reporting year, and co-existing diseases (chronic kidney disease, hypertension, diabetes, cardiac failure, and sepsis).

*AKI* acute kidney injury, *ROR* reporting odds ratio, *95% CI* 95% confidence interval, *VCM* vancomycin.

## Discussion

The JADER database analysis revealed a positive signal of acute kidney injury during teicoplanin co-administered loop diuretics. In contrast, we did not observe a significant signal of acute kidney injury in vancomycin co-administered with any type of diuretics. Since co-administration of nephrotoxic drugs is a modifiable risk factor^10^, our finding may lead to a risk reduction of acute kidney injury by avoiding co-administered loop diuretics. These findings imply that there is a need for more detailed monitoring and further studies on the concomitant use of teicoplanin and loop diuretics to prevent acute kidney injury.

Preclinical studies have shown that teicoplanin and vancomycin-induced nephrotoxicity is due to the accumulation of these drugs in the proximal tubule cells, which triggers oxidative stress and apoptosis in a concentration-dependent manner^11,12^. In particular, little is known about the mechanism of teicoplanin-induced nephrotoxicity in humans. However, several reports identified a dose-dependent manner of cell toxicity and tubular damage in a non-clinical setting^11,13^. This indicates that co-administered medications that increase exposure to glycopeptide antibiotics may increase the risk of acute kidney injury. Loop diuretics inhibit reabsorption via the sodium–potassium–chloride symporter localized in Henle’s loop, leading to a drop in glomerular filtration due to a reduction of intraglomerular pressure, nonetheless loop diuretics compensatory activate renin secretion in the macula densa to retain glomerular filtration^14,15^. The mechanism behind the increased risk of nephrotoxicity might be attributed to the inhibition of sodium–potassium–chloride symporter in afferent arteriole, which gives damage to the nephron throughout the activation of renin–angiotensin–aldosterone pathway^16^. Thus, the co-administration of loop diuretics may increase the onset of acute kidney injury associated with glycopeptide antibiotics excreted by the kidney.

A significant positive signal of acute kidney injury in teicoplanin co-administered with loop diuretics was present considering co-existing diseases such as sepsis. The meta-analysis found that teicoplanin has a preferable tolerability for nephrotoxicity compared with vancomycin^3^. Another meta-analysis found a negative correlation between the risk of nephrotoxicity associated with teicoplanin and serum albumin levels^8^. As teicoplanin is often co-prescribed with loop diuretics in patients with hypoalbuminemia due to fluid overloads, including conditions such as chronic kidney disease, hypertension, and cardiac failure, it is likely to cause acute kidney injury when used in combination with loop diuretics. In variations of diuresis, hypoalbuminemia itself increases the dose of loop diuretics to achieve favorable fluid control^17^. There is a large interindividual variability of diuretic effect in loop diuretics derived from the pharmacokinetic-pharmacodynamic relationship with gene polymorphisms of the site of action^18^. The diuretic effect might reflect the risk of acute kidney injury associated with teicoplanin.

Although we observed a significantly high aROR for acute kidney injury with vancomycin alone compared to non-target drugs, we did not detect any significant signals of acute kidney injury in vancomycin co-administered with loop diuretics. This finding is inconsistent with previous evidence suggesting that the risk of nephrotoxicity associated with vancomycin is significantly high in patients who receive loop diuretics or nephrotoxic drugs, including aminoglycosides^19–21^. This inconsistency may be due to the variations in urine output among individuals, which can be influenced by factors such as renal function, hemodynamic status, and volume status, in addition to the dose-setting of loop diuretics^22^. Furthermore, co-administration of vancomycin and loop diuretics might be avoided in terms of the high potential of nephrotoxicity. We speculate that teicoplanin is regarded as an alternative option to evade nephrotoxicity in patients with co-administered loop diuretics. As such, clinicians should exercise caution while using a combination of vancomycin and diuretics until the action mechanism is better understood.

Several limitations should be considered when interpreting these results. First, it is difficult to avoid reporting bias, including underreporting, notoriety bias, ripple effect, and the Weber effect because the database is composed of spontaneous reports. Second, information on comorbidities in JADER is biased, and not all comorbidities are documented, leading to potential confounding and interactions. In fact, the area under the curve of all models investigated in this study was approximately 0.6 (Supplementary Table 1). In the models involving teicoplanin and loop diuretics, no multicollinearity was observed; however, an interaction was detected between hypertension and the concomitant use of pharmaceutical agents. This result indicates that this study focuses on detecting interaction signals, highlighting the importance of more extensive hypothesis-validation research. Third, the JADER database does not provide the true number of cases for each drug, making it difficult to determine the absolute risk of events. Fourth, the lack of data on clinical laboratory tests (e.g., serum creatinine level) prevented accurate grading and definition of acute kidney injury, and further analysis should be adjusted for other variables, such as renal function and dose-setting. In particular, we did not use common definitions (e.g., Kidney Disease Improving Global Outcomes criteria). Fifth, we had to exclude some cases due to missing data. Sixth, it was not possible to completely evaluate the causality between acute kidney injury and medications. Seventh, there were no sufficient data on detailed medication information (e.g., dose and trough concentrations) and intensive care setting. To overcome this limitation, a large size of clinical study was warranted. Finally, data on events associated with the use of nephrotoxic medications and diuretics, including thiazide and vaptans, were limited.

In summary, our findings indicate that the co-administration of loop diuretics may have an unfavorable effect on acute kidney injury during teicoplanin. In contrast, co-administration of any diuretics did not provide significant signals of acute kidney injury associated with vancomycin. Additional studies are needed to validate our results and identify the variables that determine the synergistic and/or additive effects of loop diuretics on acute kidney injury associated with teicoplanin.

## Methods

### Study population and data collection

No informed consent was required because our analysis was based on a public database. We obtained data from JADER, which collects adverse event reports submitted to the Pharmaceuticals and Medical Devices Agency, as well as from publicly available sources of information. We downloaded data from April 2004 to August 2022 from the Pharmaceuticals and Medical Devices Agency’s website on August 26, 2022. JADER comprises the following four datasets: “DEMO” (demographic information), “DRUG” (drug administration information), “REAC” (adverse event information), and “HIST” (comorbidity information). We performed data cleaning to remove duplicate and incomplete records. Age information was stored in 10-year intervals in the “DEMO” dataset. In cases where multiple grades of severity were reported, we determined the severity of acute kidney injury based on the most severe grade recorded.

### Adverse event detection

In the present study, we used the Japanese version of the Medical Dictionary for Regulatory Activities (MedDRA), MedDRA/J ver. 25.1J. We identified acute kidney injury events using PTs in the Standardized MedDRA Query (SMQ) for “acute renal failure (narrow)” [20000003]. We extracted information, including sex, age, reporting year, co-existing diseases, co-administration of medications, and events of acute kidney injury. Co-existing diseases and adverse events were coded using preferred terms (PTs) derived from the MedDRA (Supplementary Table 2).

The target drugs were teicoplanin or vancomycin with intravenous administration and diuretics. The sub-category for target drugs was defined as glycopeptide antibiotics alone (teicoplanin or vancomycin), glycopeptide with diuretics, and non-target drugs. We categorized individual diuretics based on the Kyoto Encyclopedia of Genes and Genomes (KEGG) Drug database as follows: loop diuretics (DG01748), thiazide diuretics (DG01749), thiazide-like diuretics (DG02992, labeled as “thiazide related diuretics” on KEGG), potassium-sparing diuretics (DG01885 and D10892), and vaptans (DG01506).

### Statistical analysis

We performed disproportionality analysis using a 2 × 2 contingency table to detect signals of acute kidney injury associated with teicoplanin. The reporting odds ratio (ROR) and 95% CI were estimated using Eqs. (1) and (2) as follows:

1 $$ROR=\frac{a/c}{b/d}=\frac{ad}{bc}$$

2 $$95\% CI=exp\left[\mathrm{log}\left(ROR\right)\pm 1.96\sqrt{\frac{1}{a}+\frac{1}{b}+\frac{1}{c}+\frac{1}{d}}\right]$$

where a represents target drugs with acute kidney injury, b represents non-target drugs with acute kidney injury, c represents target drugs without acute kidney injury, and d represents non-target drugs without acute kidney injury.

We adopted the criteria for defining positive signals of drug-drug interaction based on previous studies^23–25^, which included (1) a minimum of three events of acute kidney injury in the group co-administered diuretics, (2) a lower limit of the 95% CI of the ROR in the co-administered diuretic group exceeding 1.0, and (3) higher RORs in the group co-administered diuretics than in the other index groups (i.e., teicoplanin or vancomycin alone and diuretics alone), and mutually exclusive 95% CIs.

We analyzed the impact of individual diuretics on acute kidney injury associated with teicoplanin or vancomycin using the ROR and 95% CI. The individual diuretics included loop diuretics, thiazide diuretics, thiazide-like diuretics, potassium-sparing diuretics, and vaptans. We performed a multivariate logistic regression analysis to determine the aROR by including sex, age, reporting year, and co-existing diseases as covariates based on the area under the curve. We selected co-existing diseases such as chronic kidney disease, hypertension, diabetes, cardiac failure, and sepsis as potential confounders based on clinical relevance.

All statistical analyses were conducted using JMP® Pro 16.2 software (SAS Institute Inc., Cary, NC, USA). p < 0.05 was regarded as statistically significant. Categorical data were summarized as numbers (%).

### Supplementary Information

Supplementary Tables.

## Data Availability

Data are available from the corresponding author upon reasonable request.
