# Supplementary material for: Drug–drug interaction signals between loop diuretics and teicoplanin during acute kidney injury evaluated using Japanese spontaneous adverse drug event reports
Source: Sci Rep. 2023 Aug 26;13:13989. doi: 10.1038/s41598-023-41095-4 (PMC10460414; doi:10.1038/s41598-023-41095-4)
Supplement: Supplementary file 1 — Supplementary Tables. [file 41598_2023_41095_MOESM1_ESM.docx]

**Supplementary Table 1** **AUC of the ROC curve of all multivariate logistic regression model in this study**

| **Model** | **AUC** |
| --- | --- |
| TEIC + loop diuretics | 0.62 |
| TEIC + thiazide diuretics | 0.62 |
| TEIC + thiazide-like diuretics | 0.62 |
| TEIC + potassium-sparing diuretics | 0.62 |
| TEIC + Vaptans | 0.62 |
| VCM + loop diuretics | 0.63 |
| VCM + thiazide diuretics | 0.62 |
| VCM + thiazide-like diuretics | 0.62 |
| VCM + potassium-sparing diuretics | 0.63 |
| VCM + Vaptans | 0.62 |

Abbreviations: AUC; area under the curve, ROC; receiver operating characteristic curve, TEIC; teicoplanin, VCM; vancomycin, ROR; reporting odds ratio

A multivariate logistic regression model was used to determine the adjusted ROR by introducing age, sex, reporting year, and co-existing diseases (chronic kidney disease, hypertension, diabetes, cardiac failure, and sepsis).

**Supplementary Table 2a PT list for identification of acute kidney injury events**

| **Standard MedDRA Query** | **PT code** | **PT** |
| --- | --- | --- |
| “Acute renal failure (narrow)” [20000003] | 10069339 | Acute kidney injury |
|  | 10069688 | Acute phosphate nephropathy |
|  | 10002847 | Anuria |
|  | 10003885 | Azotaemia |
|  | 10066338 | Continuous haemodiafiltration |
|  | 10061105 | Dialysis |
|  | 10078987 | Foetal renal impairment |
|  | 10018875 | Haemodialysis |
|  | 10053090 | Haemofiltration |
|  | 10049778 | Neonatal anuria |
|  | 10029155 | Nephropathy toxic |
|  | 10030302 | Oliguria |
|  | 10034660 | Peritoneal dialysis |
|  | 10072370 | Prerenal failure |
|  | 10038435 | Renal failure |
|  | 10038447 | Renal failure neonatal |
|  | 10062237 | Renal impairment |
|  | 10049776 | Renal impairment neonatal |
|  | 10081980 | Subacute kidney injury |

Abbreviations: MedDRA; Medical Dictionary for Regulatory Activities, PT; preferred term

**Supplementary Table 2b PT list used for identification of chronic kidney disease events**

| **Standard MedDRA Query** | **PT code** | **PT** |
| --- | --- | --- |
| “Chronic kidney disease (narrow)” [20000213] | 10087686 | APOL1-mediated kidney disease |
|  | 10053699 | Artificial kidney device user |
|  | 10003885 | Azotaemia |
|  | 10064848 | Chronic kidney disease |
|  | 10078095 | Chronic kidney disease-mineral and bone disorder |
|  | 10010082 | Coma uraemic |
|  | 10012660 | Diabetic end stage renal disease |
|  | 10061105 | Dialysis |
|  | 10059015 | Dialysis device insertion |
|  | 10077512 | End stage renal disease |
|  | 10083258 | Erythropoietin deficiency anaemia |
|  | 10018367 | Glomerulonephritis chronic |
|  | 10018875 | Haemodialysis |
|  | 10053090 | Haemofiltration |
|  | 10019845 | Hepatorenal failure |
|  | 10062624 | High turnover osteopathy |
|  | 10020708 | Hyperparathyroidism secondary |
|  | 10023421 | Kidney fibrosis |
|  | 10063000 | Low turnover osteopathy |
|  | 10081588 | Metabolic nephropathy |
|  | 10058116 | Nephrogenic anaemia |
|  | 10067467 | Nephrogenic systemic fibrosis |
|  | 10029159 | Nephrosclerosis |
|  | 10049630 | Oedema due to renal disease |
|  | 10034498 | Pericarditis uraemic |
|  | 10034660 | Peritoneal dialysis |
|  | 10052279 | Renal and liver transplant |
|  | 10052278 | Renal and pancreas transplant |
|  | 10087816 | Renal artery revascularisation |
|  | 10038435 | Renal failure |
|  | 10074746 | Renal replacement therapy |
|  | 10038519 | Renal rickets |
|  | 10038533 | Renal transplant |
|  | 10056609 | Uraemia odour |
|  | 10046324 | Uraemic acidosis |
|  | 10087409 | Uraemic cardiomyopathy |
|  | 10046326 | Uraemic encephalopathy |
|  | 10063709 | Uraemic gastropathy |
|  | 10077910 | Uraemic myopathy |
|  | 10046328 | Uraemic neuropathy |
|  | 10060875 | Uraemic pruritus |
|  | 10067863 | Uridrosis |

Abbreviations: MedDRA; Medical Dictionary for Regulatory Activities, PT; preferred term

**Supplementary Table 2c PT list used for identification of hypertension**

| **Standard MedDRA Query** | **PT code** | **PT** |
| --- | --- | --- |
| “Hypertension (narrow)” [20000147] | 10000358 | Accelerated hypertension |
|  | 10005732 | Blood pressure ambulatory increased |
|  | 10005739 | Blood pressure diastolic increased |
|  | 10051128 | Blood pressure inadequately controlled |
|  | 10005750 | Blood pressure increased |
|  | 10063926 | Blood pressure management |
|  | 10053355 | Blood pressure orthostatic increased |
|  | 10005760 | Blood pressure systolic increased |
|  | 10081751 | Catecholamine crisis |
|  | 10063067 | Dialysis induced hypertension |
|  | 10012758 | Diastolic hypertension |
|  | 10014129 | Eclampsia |
|  | 10057615 | Endocrine hypertension |
|  | 10015488 | Essential hypertension |
|  | 10070538 | Gestational hypertension |
|  | 10049058 | HELLP syndrome |
|  | 10020571 | Hyperaldosteronism |
|  | 10020772 | Hypertension |
|  | 10049781 | Hypertension neonatal |
|  | 10059238 | Hypertensive angiopathy |
|  | 10020801 | Hypertensive cardiomegaly |
|  | 10058222 | Hypertensive cardiomyopathy |
|  | 10077000 | Hypertensive cerebrovascular disease |
|  | 10020802 | Hypertensive crisis |
|  | 10058179 | Hypertensive emergency |
|  | 10020803 | Hypertensive encephalopathy |
|  | 10079496 | Hypertensive end-organ damage |
|  | 10020823 | Hypertensive heart disease |
|  | 10055171 | Hypertensive nephropathy |
|  | 10058181 | Hypertensive urgency |
|  | 10049079 | Labile hypertension |
|  | 10025600 | Malignant hypertension |
|  | 10025603 | Malignant hypertensive heart disease |
|  | 10026674 | Malignant renal hypertension |
|  | 10026924 | Maternal hypertension affecting foetus |
|  | 10026985 | Mean arterial pressure increased |
|  | 10052066 | Metabolic syndrome |
|  | 10067598 | Neurogenic hypertension |
|  | 10065508 | Orthostatic hypertension |
|  | 10076704 | Page kidney |
|  | 10050631 | Postoperative hypertension |
|  | 10036485 | Pre-eclampsia |
|  | 10065918 | Prehypertension |
|  | 10062886 | Procedural hypertension |
|  | 10087816 | Renal artery revascularisation |
|  | 10038464 | Renal hypertension |
|  | 10074864 | Renal sympathetic nerve ablation |
|  | 10038562 | Renovascular hypertension |
|  | 10038926 | Retinopathy hypertensive |
|  | 10039808 | Secondary aldosteronism |
|  | 10039834 | Secondary hypertension |
|  | 10084825 | Superimposed pre-eclampsia |
|  | 10078932 | Supine hypertension |
|  | 10042957 | Systolic hypertension |
|  | 10048007 | Withdrawal hypertension |

Abbreviations: MedDRA; Medical Dictionary for Regulatory Activities, PT; preferred term

**Supplementary Table 2d PT list used for identification of diabetes mellitus**

| **Standard MedDRA Query** | **PT code** | **PT** |
| --- | --- | --- |
| “Hyperglycaemia/ new onset diabetes mellitus (narrow)” [20000041] | 10087376 | Acquired generalised lipodystrophy |
|  | 10065367 | Blood 1,5-anhydroglucitol decreased |
|  | 10005557 | Blood glucose increased |
|  | 10012596 | Diabetes complicating pregnancy |
|  | 10012601 | Diabetes mellitus |
|  | 10012607 | Diabetes mellitus inadequate control |
|  | 10012631 | Diabetes with hyperosmolarity |
|  | 10077357 | Diabetic arteritis |
|  | 10012650 | Diabetic coma |
|  | 10080788 | Diabetic coronary microangiopathy |
|  | 10071265 | Diabetic hepatopathy |
|  | 10012668 | Diabetic hyperglycaemic coma |
|  | 10012669 | Diabetic hyperosmolar coma |
|  | 10012671 | Diabetic ketoacidosis |
|  | 10012672 | Diabetic ketoacidotic hyperglycaemic coma |
|  | 10012673 | Diabetic ketosis |
|  | 10074309 | Diabetic metabolic decompensation |
|  | 10081558 | Diabetic wound |
|  | 10080061 | Euglycaemic diabetic ketoacidosis |
|  | 10017395 | Fructosamine increased |
|  | 10072628 | Fulminant type 1 diabetes mellitus |
|  | 10018209 | Gestational diabetes |
|  | 10018429 | Glucose tolerance impaired |
|  | 10018430 | Glucose tolerance impaired in pregnancy |
|  | 10018478 | Glucose urine present |
|  | 10082836 | Glycated albumin increased |
|  | 10087214 | Glycated serum protein increased |
|  | 10018473 | Glycosuria |
|  | 10018475 | Glycosuria during pregnancy |
|  | 10018481 | Glycosylated haemoglobin abnormal |
|  | 10018484 | Glycosylated haemoglobin increased |
|  | 10085610 | Hepatogenous diabetes |
|  | 10020635 | Hyperglycaemia |
|  | 10087319 | Hyperglycaemic crisis |
|  | 10063554 | Hyperglycaemic hyperosmolar nonketotic syndrome |
|  | 10071394 | Hyperglycaemic seizure |
|  | 10071286 | Hyperglycaemic unconsciousness |
|  | 10056997 | Impaired fasting glucose |
|  | 10022489 | Insulin resistance |
|  | 10022491 | Insulin resistant diabetes |
|  | 10053247 | Insulin-requiring type 2 diabetes mellitus |
|  | 10023379 | Ketoacidosis |
|  | 10023388 | Ketonuria |
|  | 10023391 | Ketosis |
|  | 10023392 | Ketosis-prone diabetes mellitus |
|  | 10066389 | Latent autoimmune diabetes in adults |
|  | 10086189 | Maternally inherited diabetes and deafness |
|  | 10075980 | Monogenic diabetes |
|  | 10028933 | Neonatal diabetes mellitus |
|  | 10086425 | Neonatal hyperglycaemia |
|  | 10082630 | New onset diabetes after transplantation |
|  | 10033660 | Pancreatogenous diabetes |
|  | 10087435 | Pseudodiabetes |
|  | 10081755 | Steroid diabetes |
|  | 10067584 | Type 1 diabetes mellitus |
|  | 10067585 | Type 2 diabetes mellitus |
|  | 10072659 | Type 3 diabetes mellitus |
|  | 10057597 | Urine ketone body present |

Abbreviations: MedDRA; Medical Dictionary for Regulatory Activities, PT; preferred term

**Supplementary Table 2e PT list used for identification of cardiac failure**

| **Standard MedDRA Query** | **PT code** | **PT** |
| --- | --- | --- |
| “Cardiac failure (narrow)” [20000004] | 10063081 | Acute left ventricular failure |
|  | 10001029 | Acute pulmonary oedema |
|  | 10063082 | Acute right ventricular failure |
|  | 10007522 | Cardiac asthma |
|  | 10007554 | Cardiac failure |
|  | 10007556 | Cardiac failure acute |
|  | 10007558 | Cardiac failure chronic |
|  | 10007559 | Cardiac failure congestive |
|  | 10007560 | Cardiac failure high output |
|  | 10007625 | Cardiogenic shock |
|  | 10082480 | Cardiohepatic syndrome |
|  | 10051093 | Cardiopulmonary failure |
|  | 10068230 | Cardiorenal syndrome |
|  | 10063083 | Chronic left ventricular failure |
|  | 10063084 | Chronic right ventricular failure |
|  | 10084058 | Congestive hepatopathy |
|  | 10010968 | Cor pulmonale |
|  | 10010969 | Cor pulmonale acute |
|  | 10010970 | Cor pulmonale chronic |
|  | 10050528 | Ejection fraction decreased |
|  | 10051448 | Hepatojugular reflux |
|  | 10024119 | Left ventricular failure |
|  | 10024899 | Low cardiac output syndrome |
|  | 10049780 | Neonatal cardiac failure |
|  | 10073708 | Obstructive shock |
|  | 10037423 | Pulmonary oedema |
|  | 10050459 | Pulmonary oedema neonatal |
|  | 10076203 | Radiation associated cardiac failure |
|  | 10075337 | Right ventricular ejection fraction decreased |
|  | 10039163 | Right ventricular failure |
|  | 10060953 | Ventricular failure |

Abbreviations: MedDRA; Medical Dictionary for Regulatory Activities, PT; preferred term

**Supplementary Table 2f PT list used for identification of sepsis**

| Standard MedDRA Query | PT code | PT |
| --- | --- | --- |
| “Sepsis (narrow)” [20000234] | 10058040 | Abdominal sepsis |
|  | 10083897 | Acinetobacter sepsis |
|  | 10080434 | Actinomycotic sepsis |
|  | 10051407 | Amniotic infection syndrome of Blane |
|  | 10058873 | Anthrax sepsis |
|  | 10053840 | Bacterial sepsis |
|  | 10057847 | Biliary sepsis |
|  | 10054210 | Brucella sepsis |
|  | 10069684 | Burkholderia cepacia complex sepsis |
|  | 10070681 | Campylobacter sepsis |
|  | 10053166 | Candida sepsis |
|  | 10081740 | Capnocytophaga sepsis |
|  | 10070671 | Cerebral septic infarct |
|  | 10054213 | Citrobacter sepsis |
|  | 10078496 | Clostridial sepsis |
|  | 10057767 | Corynebacterium sepsis |
|  | 10069802 | Device related sepsis |
|  | 10014824 | Endotoxic shock |
|  | 10054219 | Enterobacter sepsis |
|  | 10054221 | Enterococcal sepsis |
|  | 10080432 | Erysipelothrix sepsis |
|  | 10015296 | Escherichia sepsis |
|  | 10058872 | Fungal sepsis |
|  | 10053588 | Group B streptococcus neonatal sepsis |
|  | 10069762 | Haematological infection |
|  | 10058875 | Haemophilus sepsis |
|  | 10054264 | Helicobacter sepsis |
|  | 10058876 | Herpes sepsis |
|  | 10074246 | Herpes simplex sepsis |
|  | 10075622 | Intestinal sepsis |
|  | 10054160 | Klebsiella sepsis |
|  | 10085989 | Leclercia bacteraemia |
|  | 10083161 | Leptospira sepsis |
|  | 10063085 | Listeria sepsis |
|  | 10027280 | Meningococcal sepsis |
|  | 10054162 | Micrococcal sepsis |
|  | 10028615 | Myocarditis septic |
|  | 10049151 | Neutropenic sepsis |
|  | 10064952 | Nocardia sepsis |
|  | 10059070 | Pelvic sepsis |
|  | 10058889 | Plague sepsis |
|  | 10054047 | Pneumococcal sepsis |
|  | 10086165 | Porphyromonas bacteraemia |
|  | 10066593 | Post procedural sepsis |
|  | 10036422 | Postpartum sepsis |
|  | 10058973 | Pseudallescheria sepsis |
|  | 10058877 | Pseudomonal sepsis |
|  | 10051739 | Pulmonary sepsis |
|  | 10084639 | SARS-CoV-2 sepsis |
|  | 10058878 | Salmonella sepsis |
|  | 10040047 | Sepsis |
|  | 10040049 | Sepsis neonatal |
|  | 10040051 | Sepsis pasteurella |
|  | 10053879 | Sepsis syndrome |
|  | 10040059 | Septic arthritis haemophilus |
|  | 10040061 | Septic arthritis neisserial |
|  | 10040063 | Septic arthritis staphylococcal |
|  | 10040064 | Septic arthritis streptobacillus |
|  | 10067323 | Septic arthritis streptococcal |
|  | 10087221 | Septic cardiomyopathy |
|  | 10086435 | Septic cerebral embolism |
|  | 10083159 | Septic coagulopathy |
|  | 10040067 | Septic embolus |
|  | 10069141 | Septic encephalopathy |
|  | 10052762 | Septic necrosis |
|  | 10056518 | Septic phlebitis |
|  | 10083093 | Septic pulmonary embolism |
|  | 10062657 | Septic rash |
|  | 10040070 | Septic shock |
|  | 10073007 | Septic vasculitis |
|  | 10058879 | Serratia sepsis |
|  | 10074481 | Shigella sepsis |
|  | 10056430 | Staphylococcal sepsis |
|  | 10054137 | Stenotrophomonas sepsis |
|  | 10048960 | Streptococcal sepsis |
|  | 10044248 | Toxic shock syndrome |
|  | 10044250 | Toxic shock syndrome staphylococcal |
|  | 10044251 | Toxic shock syndrome streptococcal |
|  | 10045470 | Umbilical sepsis |
|  | 10048709 | Urosepsis |
|  | 10071362 | Viral sepsis |
|  | 10047847 | Waterhouse-Friderichsen syndrome |
|  | 10058041 | Wound sepsis |
|  | 10072902 | Yersinia sepsis |

Abbreviations: MedDRA; Medical Dictionary for Regulatory Activities, PT; preferred term
